# A suicide gene approach using the human pro-apoptotic protein tBid inhibits HIV-1 replication

**DOI:** 10.1186/1472-6750-11-4

**Published:** 2011-01-11

**Authors:** Peter M Huelsmann, Andreas D Hofmann, Stefanie A Knoepfel, Jasmin Popp, Pia Rauch, Francesca Di Giallonardo, Christina Danke, Eva Gueckel, Axel Schambach, Horst Wolff, Karin J Metzner, Christian Berens

**Affiliations:** 1 University of Erlangen-Nuremberg, Institute of Clinical and Molecular Virology, Erlangen, Germany; 2 University of Erlangen-Nuremberg, Department Biology, Erlangen, Germany; 3 University of Zurich, University Hospital Zurich, Department of Medicine, Division of Infectious Diseases and Hospital Epidemiology, Zurich, Switzerland; 4 University of Erlangen-Nuremberg, Department of Internal Medicine III, Erlangen, Germany; 5 Hannover Medical School, Department of Experimental Hematology, Hannover, Germany; 6 Helmholtz Zentrum München, Institute of Virology, Neuherberg, Germany

## Abstract

**Background:**

Regulated expression of suicide genes is a powerful tool to eliminate specific subsets of cells and will find widespread usage in both basic and applied science. A promising example is the specific elimination of human immunodeficiency virus type 1 (HIV-1) infected cells by LTR-driven suicide genes. The success of this approach, however, depends on a fast and effective suicide gene, which is expressed exclusively in HIV-1 infected cells. These preconditions have not yet been completely fulfilled and, thus, success of suicide approaches has been limited so far. We tested truncated Bid (tBid), a human pro-apoptotic protein that induces apoptosis very rapidly and efficiently, as suicide gene for gene therapy against HIV-1 infection.

**Results:**

When tBid was introduced into the HIV-1 LTR-based, Tat- and Rev-dependent transgene expression vector pLRed(INS)_2_R, very efficient induction of apoptosis was observed within 24 hours, but only in the presence of both HIV-1 regulatory proteins Tat and Rev. Induction of apoptosis was not observed in their absence. Cells containing this vector rapidly died when transfected with plasmids containing full-length viral genomic DNA, completely eliminating the chance for HIV-1 replication. Viral replication was also strongly reduced when cells were infected with HIV-1 particles.

**Conclusions:**

This suicide vector has the potential to establish a safe and effective gene therapy approach to exclusively eliminate HIV-1 infected cells before infectious virus particles are released.

## Background

Both basic and clinical science can greatly benefit from vectors that induce cell death in a temporally and spatially controlled manner. Inducible death vectors do not only contribute to determining the precise effect of specific cells on behavior [1], development [2], and disease [3,4], they can also provide critical tools for treatment of many diverse diseases, including adoptive immune transfer, cancer therapy, atherosclerosis, stem cell transplantation, or viral infection [5-10]. Enzymes and toxins of bacterial or viral origin are frequently used to kill the targeted cells. However, they are not necessarily the best choice for all applications, due to complications that can arise from immune responses [11], cell cycle dependence [12], and bystander killing [13]. Ectopic expression of endogenous pro-apoptotic proteins is an attractive alternative, because they should be non-immunogenic, they initiate a well-established cellular program [14,15], and their target cells are frequently primed for apoptosis [16,17].

The pro-apoptotic proteins most often used in such applications are caspases [7,8,18,19] and members of the Bcl-2 family, like BimS [20], Bax [21,22], or truncated Bid (tBid; BH3 interacting domain death agonist) [23-25]. tBid is generated by cleavage of Bid through activated caspases-2 and -8, granzyme B, and cathepsins. Translocation of tBid to the mitochondria leads to the release of cytochrome c resulting in the activation of apoptosis-inducing caspases [26-28]. Transient or inducible expression of tBid leads to rapid and efficient induction of apoptosis in a variety of cell lines [23-25].

Thus, this pro-apoptotic protein represents a promising candidate for a suicide gene therapy that aims at specifically eliminating cells infected by human immunodeficiency virus type 1 (HIV-1) before infectious viruses are produced. An infection with HIV-1 is still incurable despite constant improvements to antiretroviral therapy which dramatically reduced the mortality rate of HIV-1-infected patients [29]. Unfortunately, a substantial fraction of these patients still experiences therapy failure and/or serious side effects due to the treatment. This is often accompanied by the emergence of drug-resistant viruses [30]. Thus, improving the existing approaches and finding new antiretroviral strategies remains a major challenge in the fight against HIV-1. Numerous suicide genes have already been tested, for instance, Bax [21], protein kinase R [31], caspase-3 [18], the host shut-off protein of herpes simplex virus [32], diphtheria toxin A [33,34], and barnase [35]. However, most of them were either not efficient enough and/or cell death was not induced fast enough ultimately resulting in insufficient inhibition of HIV-1 replication. The kinetics observed and the very low protein concentrations needed to induce cell death by tBid [23] would fulfill two major requirements of a successful suicide gene: Efficiency and immediacy.

Another obstacle to this approach is the basal activity of the HIV-1 LTR promoters used, which led to unspecific cell death of HIV-1 uninfected cells [34,36,37]. The new retroviral vector pLRed(INS)_2_R utilizes three distinct elements originating from HIV-1 to more stringently regulate transgene expression: i) It contains the 5' and 3' HIV-1 LTR, so that efficient gene expression requires HIV-1 Tat [38]; ii) the vector comprises the HIV-1 Rev-responsive element (RRE) leading to Rev-dependence for mRNA transport from the nucleus to the cytoplasm [39]; and iii) two HIV-1 inhibitory sequences (INS) of the gag gene are incorporated into this vector further enhancing the dependency on Rev in terms of mRNA export significantly [40]. This vector fulfills the criterion of HIV-1 specificity and, consequently, provides a safe gene therapy vector.

Here, we replaced the reporter gene dsRed in the improved HIV-1 indicator plasmid pLRed(INS)_2_R [40] with the very potent suicide protein tBid. The resulting suicide vector was highly dependent on HIV-1 Tat and Rev for target gene expression. It very efficiently and rapidly induced apoptosis in vitro before infectious viruses were released from the HIV-1 infected cell and, therefore, provides a promising gene therapy approach against HIV-1 infection.

## Results

### tBid induces efficiently and rapidly cell death in HeLa SS6 cells

**Figure 1:**
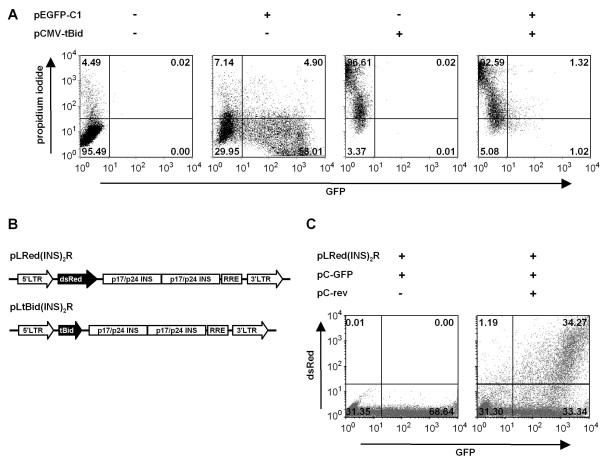
**Induction of cell death by tBid in HeLa SS6 cells and general architecture of the control vector pLRed(INS)**_**2**_**R and the suicide vector pLtBid(INS)**_**2**_**R**. (**A**) Hela SS6 cells were transfected with pCMV-tBid and/or pEGFP-C1. 24 hours after transfection, cell death and GFP expression were determined by FACS analysis. Shown is a representative example of three independent experiments. Percentages of GFP-positive, PI-positive and GFP + PI-positive cells are given for each quadrant. (**B**) Schematic representation of pLRed(INS)_2_R and pLtBid(INS)_2_R. p17/p24 INS = INS-containing region from *gag*; RRE = Rev-responsive element. (**C**) FACS analysis of dsRed expression from pLRed(INS)_2_R without (left) and with Rev (right) in HeLa-Tat cells constitutively expressing Tat. A plasmid, driving GFP expression under the control of the CMV promoter, was transfected as a transfection/expression control (modified from reference [40]).

To estimate the susceptibility of the cell line HeLa SS6 towards induction of cell death by the human pro-apoptotic protein tBid, we transiently co-transfected these cells with the plasmid pCMV-tBid which constitutively expresses tBid under control of a CMV-promoter, and with pEGFP-C1 allowing us to determine transfection efficiencies by measuring green fluorescence. 24 hours after transfection, GFP was expressed in 63% of the HeLa SS6 cells (Figure 1A). This number increased to >90% 48 hours after transfection (data not shown).

tBid induced cell death in ~97% of the HeLa SS6 cells within 24 hours after transfection with pCMV-tBid and in ~94% of pCMV-tBid/pEGFP-C1 co-transfected cells (Figure 1A). Similar results were obtained in two additional independent experiments. Interestingly, the co-transfected HeLa SS6 cells died before GFP expression was detected indicating very rapid induction of cell death, in agreement with published data for HeLa cells [23]. Time-course experiments confirmed this observation: Light microscopic analyses showed apoptotic HeLa SS6 cells already 10 hours after transfection (data not shown).

### Induction of cell death by tBid in the suicide vector pLtBid(INS)_2_R strongly depends on the presence of both HIV-1 Tat and Rev

**Figure 2:**
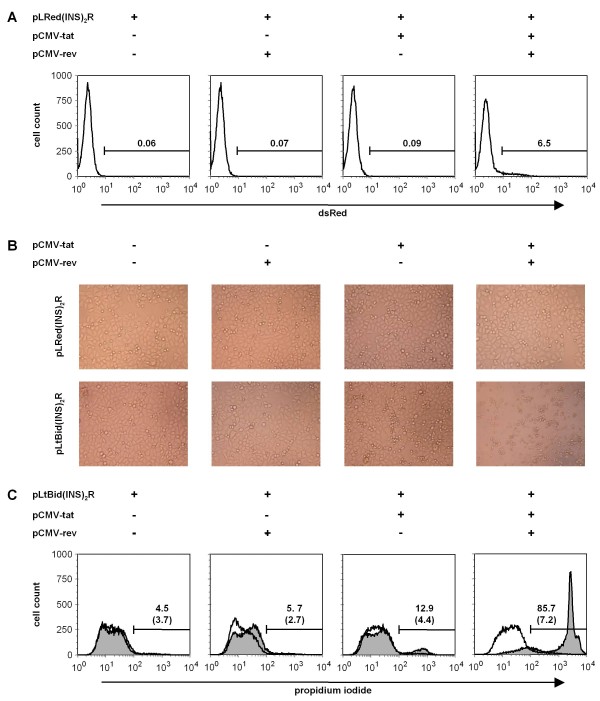
**Induction of cell death by tBid expressed by the suicide vector pLtBid(INS)**_**2**_**R and its dependency on the presence of HIV-1 Tat and Rev**. Shown are representative examples of three independent experiments. (**A**) HeLa SS6 cells were transfected with the control vector pLRed(INS)_2_R alone or in addition with pCMV-rev and/or pCMV-tat. 24 hours after infection, expression of dsRed was measured by FACS analysis. Percentages of dsRed-positive cells are given. (**B/C**) HeLa SS6 cells were transfected with pLRed(INS)_2_R or the suicide vector pLtBid(INS)_2_R alone or in addition with pCMV-rev and/or pCMV-tat. 24 hours after infection, cell death was determined by light microscopy (**B**) and PI-staining followed by FACS analysis (**C**). Percentages of dead cells are given for cells transfected with pLtBid(INS)_2_R and in parenthesis for cells tranfected with pLRed(INS)_2_R. Data obtained by transfection with the control vector pLRed(INS)_2_R are depicted in white, the results of transfections with the suicide vector pLtBid(INS)_2_R are shown in grey.

First, we characterized the control vector pLRed(INS)_2_R (Figure 1B) in transiently transfected HeLa SS6 cells or in HeLa-Tat cells, constitutively expressing the HIV-1 Tat protein. DsRed was not expressed in the absence of Tat and Rev or in the presence of only one of both viral proteins (Figures 1C and 2A). Co-transfection of HeLa SS6 cells with the plasmids pLRed(INS)_2_R, pCMV-tat and -rev led in 6.5% of the cells to a >10-fold induction of dsRed fluorescence after 24 hours (Figure 2A), this number further increased to >40% during the next 24 hours (data not shown). In HeLa cells stably expressing the HIV-1 Tat protein, dsRed fluorescence increased up to three orders of magnitude in the presence of both Tat and Rev (Figure 1C) [40]. As already observed in pEGFP-C1-transfected HeLa SS6 cells, it takes about 48 hours to reach sufficient dsRed expression. Next, we determined the number of dead cells in HeLa SS6 cells transfected with the control vector pLRed(INS)_2_R alone or in combination with pCMV-tat and/or -rev. All cells transfected displayed similar morphology (Figure 2B, top row of panels), and the number of dead cells determined by propidium iodide staining was in the range of 2.7-7.2% and, thus, similar in all transfections (Figure 2C).

We then replaced the reporter gene dsRed with tBid to generate the suicide vector pLtBid(INS)_2_R (Figure 1B). HeLa SS6 cells were transfected with this vector and co-transfected with pCMV-tat and/or -rev. 24 hours after transfection, light microscopic analysis showed that the morphology of the transfected cells represented living cells in all transfections containing the suicide vector pLtBid(INS)_2_R alone or in combination with either pCMV-tat or -rev. However, co-transfection of both pCMV-tat and -rev together with the suicide vector pLtBid(INS)_2_R rapidly induced microscopic signs of cell death like cell shrinkage and rounding (Figure 2B, lower row of panels). Cell death was also quantified by staining the cells with propidium iodide 24 hours after transfection. 4.5% of the cells were dead in cell cultures transfected only with the suicide vector pLtBid(INS)_2_R, a number similar to the control transfections. Thus, induction of cell death was not observed in the absence of Tat and Rev despite using the very sensitive HeLa SS6 cell line. We also did not observe any increase in cell death induction 48 or 72 hours after transfection (data not shown). Expression of Rev alone did not lead to induction of cell death by tBid. Here, only 5.7% of the cells stained positive with propidium iodide (Figure 2C). The presence of Tat moderately induced cell death in the absence of Rev (13%, Figure 2C). Taken together, these results show that this vector is not leaky. Only expression of both HIV-1 regulatory proteins Tat and Rev strongly induced cell death in co-transfected HeLa SS6 cells. Here, 86% of the cells were dead 24 hours after transfection (Figure 2C). At this same time point, only 6.5% of the cells were dsRed-positive when co-transfected with the control vector pLRed(INS)_2_R and pCMV-tat plus -rev indicating that very low amounts of tBid are sufficient to induce cell death.

### Co-transfection of pLtBid(INS)_2_R and pNL4-3-Nef-IRES-GFP leads to rapid induction of cell death before virus particles are produced

**Figure 3:**
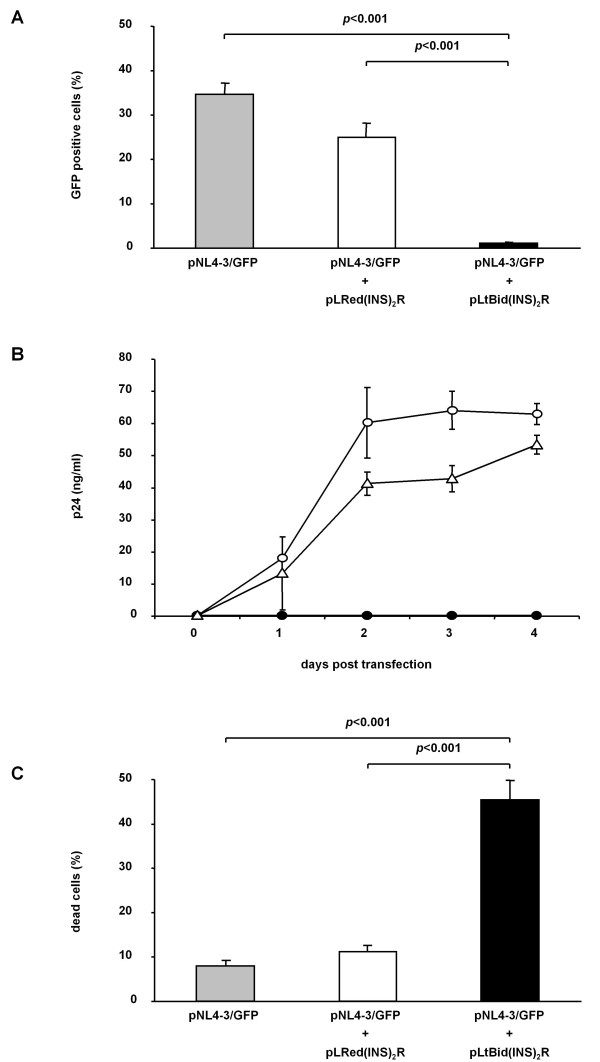
**Co-transfection of pLtBid(INS)**_**2**_**R and pNL4-3/GFP leads to rapid induction of cell death before infectious viruses are produced**. Data represent the means and standard deviations of three independent experiments. *p*-values <0.001 indicate the highly significant impact of tBid on the inhibition of virus production and the induction of cell death. (**A**) HeLa SS6 cells were transfected with pNL4-3/GFP alone or co-transfected with pNL4-3/GFP and either pLRed(INS)_2_R or pLtBid(INS)_2_R. 24 hours after transfection, viral gene expression was determined by analyzing GFP expression using FACS. (**B**) HeLa SS6 cells were transfected with pLRed(INS)_2_R (open triangles) or pLtBid(INS)_2_R (black circles) together with pNL4-3/GFP or transfected with pNL4-3/GFP alone (open circles). Release of viral particles was monitored daily for 4 days by p24 antigen ELISA. (**C**) Determination of cell death in HeLa SS6 cells transfected only with pNL4-3/GFP or co-transfected with pLRed(INS)_2_R and pNL4-3/GFP or with pLtBid(INS)_2_R and pNL4-3/GFP, 24 hours after transfection. Percentages of DCD-stained cells are depicted.

To investigate whether HeLa SS6 cells transfected with the suicide vector pLtBid(INS)_2_R undergo cell death before infectious HIV-1 virions are generated and released, we co-transfected cells with the suicide vector pLtBid(INS)_2_R and the HIV-1 full-length clone pNL4-3/GFP. The latter construct contains a GFP reporter gene within the open reading frame for Nef, ensuring that GFP is expressed together with Tat and Rev early in the HIV-1 life cycle. 27 ± 3.3% of the HeLa SS6 cells co-transfected with pNL4-3/GFP and the control vector pLRed(INS)_2_R expressed GFP 24 hours after transfection in three independent experiments. These transfection efficiencies are similar to control experiments performed with a GFP-expressing plasmid of approximately the same size as pNL4-3/GFP (~15 kb) (data not shown). In contrast, only 1.0 ± 0.3% of the HeLa SS6 cells co-transfected with pNL4-3/GFP and the suicide vector pLtBid(INS)_2_R expressed GFP 24 hours after transfection suggesting that cell death is induced in the co-transfected HeLa SS6 cells before green fluorescence becomes detectable. The approximately 28-fold difference in the amount of GFP-positive cells between both transfections is highly significant with a p-value <0.001 (Figure 3A). Transfections using only pNL4-3/GFP revealed similar results as co-transfections with the control vector pLRed(INS)_2_R and pNL4-3/GFP (Figure 3A). We also collected cell-free supernatant daily for 4 days after transfection and analyzed the production of virus particles by determining the content of viral p24 in the supernatant. In the presence of the control vector pLRed(INS)_2_R, the replication kinetics of HIV-1 NL4-3 were similar to those observed in HeLa SS6 cells only transfected with pNL4-3/GFP (Figure 3B). However, we were not able to detect p24 in any supernatant from a cell culture co-transfected with the suicide vector pLtBid(INS)_2_R and pNL4-3/GFP within fours days after co-transfection agreeing with the results obtained by measuring GFP expression in those cells.

To determine if the inhibition of virus replication is due to the rapid induction of cell death, we measured the percentage of dead cells 24 hours after transfection with pNL4-3/GFP or co-transfections with pNL4-3/GFP and either the control vector pLRed(INS)_2_R or the suicide vector pLtBid(INS)_2_R. Cells were stained with the dead cell discrimination reagent. On average, 8.1 ± 1.2% and 11 ± 1.5% of the cells transfected with pNL4-3/GFP or co-transfected with pNL4-3/GFP and the control vector pLRed(INS)_2_R, respectively, were dead 24 hours after transfection (Figure 3C). A strongly significant increase in the percentage of dead cells was observed in HeLa SS6 cells 24 hours after co-transfection with pNL4-3/GFP and the suicide vector pLtBid(INS)_2_R (average: 45 ± 4.5%, p < 0.001, Figure 3C). This clearly demonstrates that the expression of Tat and Rev from transfected HIV-1 DNA induces cell death rapidly and efficiently enough to prevent the production of new virus particles.

### Inhibition of HIV-1 NL4-3 replication in pLtBid(INS)_2_R-CD4 transfected HeLa SS6 cells

**Figure 4:**
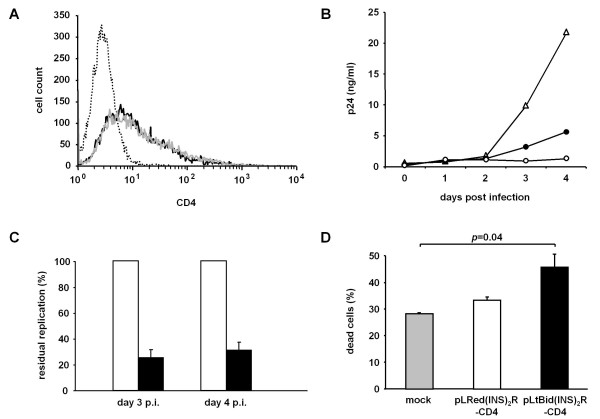
**Inhibition of HIV-1 NL4-3 replication in pLtBid(INS)**_**2**_**R-CD4 transfected HeLa SS6 cells**. HeLa SS6 cells were transfected with the control vector pLRed(INS)_2_R-CD4 or the suicide vector pLtBid(INS)_2_R-CD4. 36 hours after transfection, HeLa SS6 cells were infected with HIV-1 NL4-3. The data shown are from two independent experiments, each performed in duplicate. (**A**) CD4 expression was determined by FACS analysis 24 hours after infection with HIV-1 NL4-3 in mock transfected (dotted line), pLRed(INS)_2_R-CD4 transfected (black line), and pLtBid(INS)_2_R-CD4 transfected (grey line) HeLa SS6 cells. (**B**) Release of viral particles was determined by p24-ELISA using cell-free supernatants of HIV-1 NL4-3 infected HeLa SS6 cells transfected with pLRed(INS)_2_R-CD4 (open triangles), pLtBid(INS)_2_R-CD4 (black circles), or mock controls (open circles). Shown is one representative assessment. (**C**) Depicted is the residual replication of HIV-1 NL4-3 at days 3 and 4 post infection in pLtBid(INS)_2_R-CD4 transfected HeLa SS6 cells (black) as measured by p24 antigen ELISA, background subtracted, and normalized to p24 values obtained in pLRed(INS)_2_R-CD4 transfected HeLa SS6 cells (white). (**D**) Determination of cell death in HeLa SS6 cells in mock transfected (grey), pLRed(INS)_2_R-CD4 transfected (white), and pLtBid(INS)_2_R-CD4 transfected (black bar) cells 48 h after infection with HIV-1 NL4-3. Percentages of DCD-stained cells are depicted.

Next, we investigated the functionality of our suicide vector pLtBid(INS)_2_R in the context of infectious HIV-1 particles. We introduced a CMV-expressed cDNA for the HIV-1 receptor CD4 into the suicide vector pLtBid(INS)_2_R obtaining pLtBid(INS)_2_R-CD4. We transfected HeLa SS6 cells, which do not physiologically express the CD4 receptor with pLtBid(INS)_2_R-CD4. This guarantees that only cells containing the suicide vector would be permissive for HIV-1 infection, thereby eliminating the possibility of virus replication in HeLa SS6 cells only transfected with a CD4 expression plasmid if a co-transfection approach using separate plasmids would have been used. pLRed(INS)_2_R-CD4 was constructed as control vector. HeLa SS6 cells were infected with HIV-1 NL4-3 36 hours after transfection and viral p24 was measured daily in cell-free supernatants. To control for transfection efficiency and consistency, CD4 expression was measured 24 hours after infection by FACS analysis. pLRed(INS)_2_R-CD4- and pLtBid(INS)_2_R-CD4 transfected HeLa SS6 cells expressed similar levels of CD4 (Figure 4A). HIV-1 NL4-3 did not replicate in mock-transfected or untransfected HeLa SS6 cells (Figure 4B; data not shown). HIV-1 NL4-3 replication was inhibited by 75 ± 6.1% on day 3 and 69 ± 6.4% on day 4 after infection compared to virus replication in HeLa SS6 cells transfected with the control vector pLRed(INS)_2_R-CD4 (Figure 4B,C). We also quantified the amount of dead cells 48 hours after infection, i.e., 84 hours after transfection. 28 ± 0.5% of the mock-transfected and 33 ± 1.3% of the pLRed(INS)_2_R-CD4 transfected HeLa SS6 cells were dead (Figure 4D). These percentages of dead cells are higher compared to the amount of dead cells in our previous HeLa SS6 transfections using pLRed(INS)_2_R and pNL4-3/GFP. This is mainly due to the different time points chosen to measure the proportion of dead cells (24 versus 84 hours after transfection). An even higher increase in the number of dead cells was observed in pLtBid(INS)_2_R-CD4 transfected HeLa SS6 (46 ± 5.0%, Figure 4D) suggesting that those cells were also dying due to the induction of tBid expression by HIV-1 NL4-3. This high amount of dead cells was significantly different compared to mock-transfected cells (p = 0.04); the difference was not significant compared to pLRed(INS)_2_R-CD4-transfected HeLa SS6 cells (p = 0.08, Figure 4D). However, the latter is caused by cytopathic effects resulting from the HIV-1 infection itself, which would also explain the higher amount of dead cells in pLRed(INS)_2_R-CD4 transfected HeLa SS6 cells compared to mock-transfected cells.

## Discussion

Despite several set-backs, the development of suicide vector systems can still be a promising approach for gene therapy of HIV-1 infection [5,41]. They can be used to reduce viral burden by killing infected, virus-producing cells [9,21] and to target and eliminate the reservoir of latent HIV-1 [5]. They might even be used to modify stem cells prior to their transplantation into the patient [42,43]. These booby-trapped hematopoietic cells would commit suicide upon infection by HIV-1, thereby interrupting the viral replicative cycle and reducing viral load. However, for such an approach to function, several requirements have to be fulfilled. First, the suicide vector must be strongly HIV-1 dependent without compromising the rapid induction of the suicide gene by the viral proteins. Second, the suicide gene itself has to be fast and efficient in its induction of cell death, to ensure target cell elimination before infectious virus particles are generated. Third, the suicide gene should not be immunogenic. Fourth, the suicide system has to be delivered efficiently to the HIV-1 target cells. So far, no published system fulfills all of these necessities. In our proof-of-concept study, we addressed the first three requirements.

The suicide vector pLtBid(INS)_2_R contains the HIV-1 LTRs, thus, expression of tBid depends on HIV-1 Tat. However, the HIV-1 LTR alone would lead to unspecific expression of tBid as frequently observed using solely HIV-1 LTR-based vector systems [21,32,44]. The RRE present in the suicide vector pLtBid(INS)_2_R reduces this leakiness, because expression now depends on both Tat and Rev [9,33,34,45]. In addition, the suicide vector pLtBid(INS)_2_R comprises two INS-containing regions of the HIV-1 gag gene. They increase the dependency on Rev, thereby reducing basal activity even further [40,46,47]. We tested the control vector pLRed(INS)_2_R in HeLa SS6 cells showing that the expression of dsRed is strongly dependent on the simultaneous presence of Tat and Rev. We observed no leakiness of the suicide vector pLtBid(INS)_2_R in the very sensitive cell line HeLa SS6 transfected only with pLtBid(INS)_2_R or co-transfected with pCMV-rev. The presence of Tat alone slightly induced the expression of tBid followed by induction of cell death in a small number of cells. However, only the presence of both Tat and Rev significantly enhanced the induction of cell death introduced by tBid showing that the system is not leaky and that it strongly depends on the HIV-1 proteins Tat and Rev. It is promising that no leakiness was observed in cells stably transfected with the control vector pLRed(INS)_2_R for more than 3 months. In addition, dsRed expression was still inducible by HIV-1 Tat and Rev after 3 months [48].

The human pro-apoptotic protein tBid is the effector molecule of our suicide vector pLtBid(INS)_2_R. tBid fulfills several qualifications of a suitable suicide protein: i) cell death is induced within hours after expression of tBid, ii) tBid is efficient in very low concentrations [23], iii) it is not immunogenic as, for instance, viral suicide proteins [11,32], and iv) it has been successfully applied as suicide protein in a gene therapeutic approach where breast cancer cells were specifically killed by tBid in vitro [24]. We observed rapid induction of cell death in HeLa SS6 cells within 24 hours after transfection of pCMV-tBid. Similar kinetics and efficacies had previously been shown in several cell lines [23,25,49] including Jurkat T lymphocytes [50].

We tested the cell death inducing properties of our suicide vector pLtBid(INS)_2_R with respect to HIV-1 using different experimental approaches. First, we co-transfected cells with the suicide vector pLtBid(INS)_2_R and the full-length HIV-1 clone pNL4-3/GFP. A few hours after transfection, before infectious HIV-1 particles were generated, the cells started to die. Here, no release of virus particles was observed as revealed by p24 ELISA. Second, cells were transfected with the suicide vector pLtBid(INS)_2_R-CD4 and infected with infectious HIV-1 NL4-3. Virus replication was strongly reduced and the amount of dead cells increased compared to HeLa SS6 cells transfected with the control vector pLRed(INS)_2_R-CD4. Although we have not measured tBid directly in dying cells due to the very low amounts of tBid sufficient to induce apoptosis [23,49], which makes it very difficult to detect tBid with conventional detection methods, we are convinced that tBid expression induced cell death. In all our experiments, we used the isogenic vector pLRed(INS)_2_R as control and we never observed any indication of increased cell death induced by dsRed.

In contrast to the co-transfection experiments using pNL4-3/GFP, the inhibition of virus particle production was incomplete when pLtBid(INS)_2_R-CD4 transfected HeLa SS6 cells were infected with HIV-1 NL4-3. This is not due to the replacement of nef by gfp in pNL4-3/GFP, because we obtained similar results using pNL4-3 (data not shown). Several possible reasons could explain this observation. Residual replication might be due to the long CD4 half-life of approximately 20-24 hours on the cell surface [51,52] when rapidly dividing cells lose the transiently transfected suicide vector pLtBid(INS)_2_R-CD4. We have observed that transiently transfected plasmids were not detectable anymore after a few cell divisions in HeLa SS6 cells (unpublished observations). This would lead to the presence of cells which do not contain the suicide vector pLtBid(INS)_2_R-CD4, but still exhibit sufficient amounts of CD4 to promote HIV-1 entry and replication. It is known for a T cell-line adapted HIV-1 strain like NL4-3 that low CD4 expression does not impair virus replication [53]. It has also been described that cells lacking the CCR5 or CXCR4 receptor can be rendered CCR5^+ ^or CXCR4^+ ^by uptake of membrane-derived microparticles from CCR5- or CXCR4-positive cells [54]. There is evidence that CD4^+ ^cells undergoing programmed cell death shed microparticles that carry CD4 [55], offering another possibility of how co-cultured cells not expressing the CD4 antigen themselves could become infected after CD4 uptake.

The incomplete replication suppression might also be caused by differences in the levels and kinetics of HIV-1 Tat and Rev expression in the more physiological conditions using infectious virus particles [56,57]. A critical threshold level of Rev is required for highly productive HIV-1 infection [58]. In contrast to the HIV-1 genome, pLtBid(INS)_2_R-CD4 contains a duplication of the INS region. In the absence of Rev, gene expression of gag/pol and env mRNA is not completely inhibited in the context of wild-type HIV-1 [59], but this INS duplication results in enhanced suppression of gene expression. Thus, it might be that a threshold level of Rev for sufficient expression of tBid was not reached in all cells by the infection. To address this concern, we are currently focusing on improving the pro-apoptotic properties of tBid by (i) introducing mutations that eliminate ubiquitin acceptor sites and thereby improve tBid's intracellular stability [60] and by (ii) modifying its N-terminal sequence to allow myristoylation of tBid, which promotes targeting to mitochondria and enhances pro-apoptotic activity in cell culture [61]. A similar low efficiency was recently described for replication-competent wild-type HIV-1 virions compared to single-cycle VSV-G pseudotyped HIV-1 virus particles in another investigation of a potential Rev-dependent suicide vector [9].

## Conclusions

In summary, we have shown in three independent experimental settings that the induction of cell death by tBid in the context of our suicide vector pLtBid(INS)_2_R is not leaky, occurs very rapidly, and is highly dependent on HIV-1. Furthermore, the production of virus particles and virus replication is substantially inhibited in cells transfected with the suicide vectors pLtBid(INS)_2_R/-CD4. Thus, this vector system shows promise as a gene therapy vector.

## Methods

### Plasmid constructions

The cDNA of tBid was cloned into pEGFP-C1 (Invitrogen, Karlsruhe, Germany) by replacing GFP by tBid, which was isolated from the plasmid pWHE171 [23] to obtain the expression plasmid pCMV-tBid. For the construction of the suicide vector, pLRed(INS)_2_R [40] and pCMV-tBid were restricted with NheI and MscI or EcoRV (New England Biolabs), respectively. tBid was ligated into the vector replacing dsRed. To obtain the correct amino acid sequence of tBid, this plasmid was restricted with NheI and BssHII (NEB) and a linker was added consisting of the oligonucleotides ptBid-linker1 5'-CGCGCGGGCCGG-3' and ptBid-linker2 5'-CTAGCCGGCCCG-3' (biomers.net, Ulm, Germany). This vector is referred to as pLtBid(INS)_2_R.

For including the HIV-1 receptor CD4 in the suicide vector pLtBid(INS)_2_R and the control vector pLRed(INS)_2_R, the open reading frame of the human CD4 mRNA was amplified from cDNA of HeLa CD4^+ ^cells, kindly provided by D. Kabat (Oregon Health & Science University, Portland), using the oligonucleotides CD4 81 Hind III 5'- CGAATTCAAGCTTCAGAGGCCCTGCCATTTCTG-3' and CD4 1700 rc Xho I 5'- CATAGACTCGAGCACTCAACCAGTGAAGCCGG-3' (biomers.net, restriction sites are underlined). The CD4 amplicon and the expression vector pcDNA3.1 (Invitrogen) were restricted with HindIII and XhoI and ligated. The CMV-promoter-CD4 cassette was subcloned into the suicide vector pLtBid(INS)_2_R and the control vector pLRed(INS)_2_R by respective restrictions with NotI and subsequent ligations. These vectors are referred to as pLtBid(INS)_2_R-CD4 and pLRed(INS)_2_R-CD4.

pNL4-3/GFP (contains GFP within the nef ORF) was kindly provided by F. Kirchhoff (Institute of Virology, Ulm, Germany), pCMV-tat by B. Ensoli (National AIDS Center, Rome, Italy), and pCMV-rev is a gift from J. Hauber (Heinrich-Pette Institute, Hamburg, Germany). All vector sequences were confirmed by sequencing.

### Cells and transfections

HeLa SS6 cells were maintained in Dulbecco's modified Eagle medium (DMEM, Invitrogen) supplemented with 10% heat-inactivated fetal bovine serum (Invitrogen), 170 mM penicillin and 40 mM streptomycin. Cells were transfected using Lipofectamine 2000 (Invitrogen) according to the manufacturer's instructions. Briefly, cells were plated in 6- or 12-well cell culture plates. When ≥90% confluence was reached, cells were transfected with plasmids in a total amount of 4-6 μg or 1.5-3 μg per 6- or 12-well, respectively. Co-transfections were carried out with equal amounts of each plasmid. Light- and fluorescence microscopy was performed using an AxioVert 200 microscope (Zeiss).

### FACS analysis of cell death and reporter gene expression

To analyze cell death, propidium iodide (Merck) was used for all staining of non-infected cells. For safety reasons, cells potentially infected with HIV-1 were stained with the "dead cell discriminator (DCD) kit" (Miltenyi Biotec), which includes a cell fixation step according to the manufacturer's instructions. CD4 expression was analyzed by FACS using the murine anti-CD4 antibody SK3-phycoerythrin (Becton Dickinson). FACS analyses were performed using a FACSCalibur (Becton Dickinson). GFP- and dsRed expression was also determined by FACS.

### Viruses and infections

HIV-1 NL4-3 virus stock was generated and characterized as previously described [62]. Cells were infected 36 hours after transfection with pLtBid(INS)_2_R-CD4 or pLtBid(INS)_2_R-CD4 with a multiplicity of infection (MOI) of 0.01. Two hours after infection, cells were washed twice with PBS and further cultivated with fresh medium. Cell-free supernatants of cell cultures transfected with pNL4-3/GFP or infected with HIV-1 NL4-3 were collected daily after infection and virus production was estimated by an in-house p24 ELISA with a detection limit of 0.125 ng/ml as previously described [63].

### Statistical analysis

Differences in the induction of apoptosis, the expression of the reporter genes dsRed and GFP, or virus replication were tested for statistical significance by using a two-tailed Student's *t*-test. A p-value of <0.05 was considered a significant difference between two groups, a p-value below 0.001 highly significant.

## List of abbreviations used

CMV: cytomegalovirus; DCD: dead cell discrimination; DMEM: Dulbecco's modified Eagle's medium; EGFP: enhanced green fluorescent protein; ELISA: Enzyme Linked Immunosorbent Assay; FACS: Fluorescence Activated Cell Sorter; HIV: human immunodeficiency virus; INS: inhibitory sequences; LTR: long terminal repeat; ORF: open reading frame; RRE: Rev responsive element; VSVG: Vesicular Stomatitis Virus Glycoprotein

## Authors' contributions

PMH, KJM, and CB conceived and designed the study. PMH, ADH, SAK, and PR carried out all the collection of the data and analyzed and interpreted them together with KJM and CB. JP, FdG, CD, EG, AS, and HW participated in the characterization of the apoptotic effect of tBid in cells. AS and HW provided new reagents. PMH, ADH, KJM, and CB prepared the manuscript. All authors read and approved the final manuscript.

## Note

This work was presented in part at the Deutsch-österreichischer AIDS Kongress, 27-30 June 2007, Frankfurt, Germany (abstract F.24); 14. Jahrestagung der Deutschen Gesellschaft für Gentherapie, 18-20 July 2007, Heidelberg, Germany (abstract); and 3. European Congress of Virology, 1-5 September 2007, Nuremberg, Germany (abstract GET006).
